# Mechanism and Function of the Catch State in Molluscan Smooth Muscle: A Historical Perspective

**DOI:** 10.3390/ijms21207576

**Published:** 2020-10-14

**Authors:** Haruo Sugi, Tetsuo Ohno, Masamichi Moriya

**Affiliations:** 1 Department of Physiology, Teikyo University School of Medicine, Tokyo 173-8605, Japan; 2 Department of Sports Medicine, Teikyo Heisei University, Chibaken 290-0193, Japan; te269ohno@docomo.ne.jp (T.O.); m.moriya@thu.ac.jp (M.M.)

**Keywords:** catch state, *Mytilus*, anterior byssus retractor muscle, twitchin

## Abstract

Molluscan smooth muscles exhibit the catch state, in which both tension and resistance to stretch are maintained with very low rates of energy consumption. The catch state is studied mainly on the anterior byssus retractor muscle (ABRM) of a bivalve molluscan animal, *Mytilus,* which can easily be split into small bundles consisting of parallel fibers. The ABRM contracts actively with an increase in the intracellular free Ca ion concentration, [Ca^2+^]i, as with all other types of muscle. Meanwhile, the catch state is established after the reduction of [Ca^2+^]i to the resting level. Despite extensive studies, the mechanism underlying the catch state is not yet fully understood. This article briefly deals with (1) anatomical and ultrastructural aspects of the ABRM, (2) mechanical studies on the transition from the active to the catch state in the isotonic condition, (3) electron microscopic and histochemical studies on the intracellular translocation of Ca ions during the transition from the active to the catch state, and (4) biochemical studies on the catch state, with special reference to a high molecular mass protein, twitchin, which is known to occur in molluscan catch muscles.

## 1. Introduction

Molluscan animals constitute the phylum with the greatest range of diversity in gross anatomical forms, motility and behavior. At one extreme are fast-moving intelligent octopuses and squids, while at the other extreme are slowly moving bivalves. Although most types of molluscan muscles still await investigation, the only exception is a smooth muscle constituting part of the adductor muscle of bivalve molluscan animals. It is called the catch muscle, and has attracted the attention of investigators over many years, because it exhibits long-lasting tonic contraction with an extremely low rate of energy consumption [1]. The catch state in bivalve molluscs provides an effective means of keeping their shells closed to resist against external forces applied by predators. Due to technical difficulties in preparing adductor muscle preparations, the anterior byssal retractor muscle (ABRM) of a common mussel, *Mytilus*, has been used most frequently to study the catch state because of the following advantages: (1) the ABRM consists of parallel homogeneous muscle fibers, and is therefore suitable for mechanical experiments; (2) it is easily detached from the animal with the byssal organ and piece of shell attached to each end, and can be dissected into small fiber bundles (diameter, <1 mm); and (3) the bivalve *Mytilus* can be obtained at every seashore in the world. The catch mechanism in the ABRM works to keep the animal attached to the rock via the byssal organ, working against strong sea waves. This article intends to give a concise historical perspective of research work on the mechanism underlying the catch state, which is expected to have mechanisms in common with tonic contraction in vertebrate vascular smooth muscle, known as the latch state [2].

## 2. Contractile Apparatus of the ABRM

Like all other kinds of muscle, ABRM fibers contain both the thick and the thin filaments. The thin filament is about 10 µm in length, and consists of double helically arranged actin monomers. In all types of muscle, contractile activity is controlled by changes in intracellular Ca^2+^ concentration (pCa); the value of pCa is >9 in the relaxed state, and >6 in the fully activated state. The thin filament contains Ca^2+^-binding proteins [3,4,5,6], which may be involved in controlling mechanical activity, as with vertebrate skeletal muscle. Many thin filaments are anchored on opposite sides of spindle-shaped dense bodies, which are occasionally attached to the cell membrane at the tapered fiber end to transmit force along their entire length. The myosin filament is ~25 µm in length, and up to 75 nm in width, with tapered ends [7,8,9]. The huge size of the thick filament is associated with the presence of a protein, paramyosin, which forms the core of the thick filament. In the thick filament, myosin molecules are bound around the paramyosin core, so that myosin heads extend laterally to interact with the thin filaments [10]. The myosin molecule consists of two heavy chains and two pairs of light chains [11]. The EDTA light chain has been shown to serve as a Ca^2+^-binding protein regulating mechanical activity in molluscan smooth muscles [12,13]. The presence of the Ca^2+^-binding protein in both thick and thin filaments seems to indicate that the mechanical activity of the ABRM fibers may be controlled by both actin-based and myosin-based regulatory mechanisms.

## 3. Physiological Characteristics of the Active and the Catch States in the ABRM

**Figure 1:**
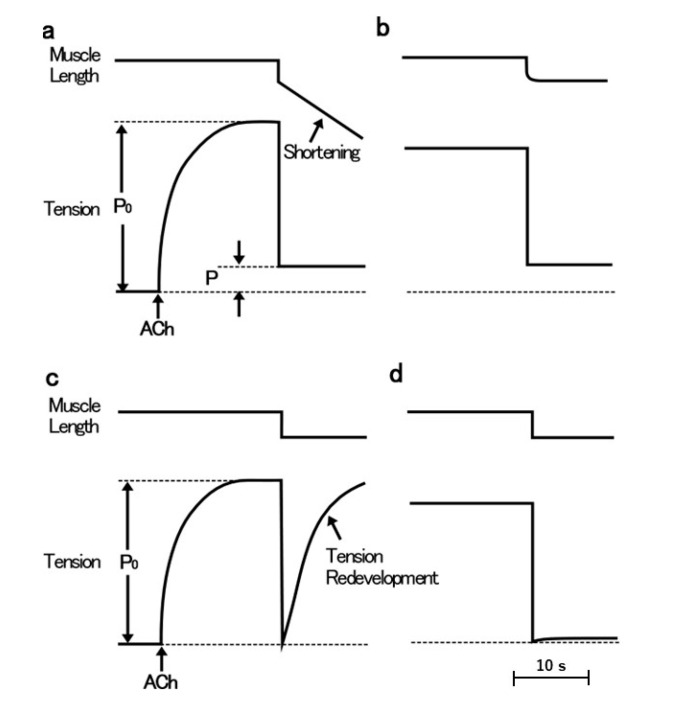
Schematic diagrams showing mechanical responses of the ABRM during the active tension development (**a**,**c**) and during the catch state (**b**,**d**). Upper traces represent changes in fiber length, while lower traces represent changes in tension. The ABRM is first stimulated by ACh (0.1 mM), and after the maximum isometric tension (Po) is developed, it is subjected to a reduction in load from Po to P < Po (**a**), or subjected to a quick release (magnitude, 5% of slack length, Lo) (**c**). When the ABRM is put into the catch state after removal of ACh, it no longer shortens after a reduction of load (**b**), and no longer redevelops tension after a quick release (**d**).

**Figure 2:**
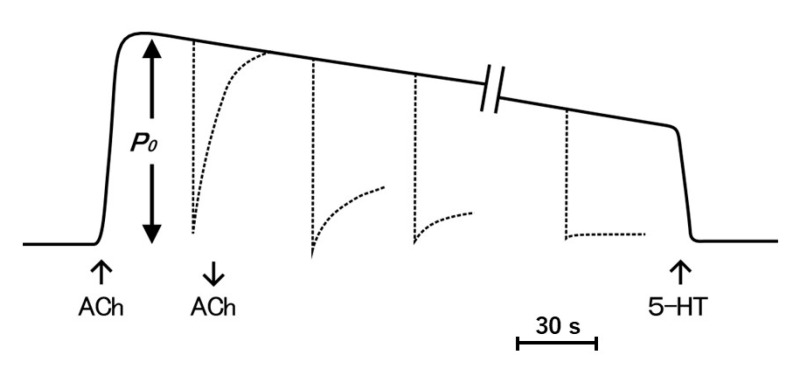
Diagram of tension record illustrating the transition from the active to the catch state. The ABRM is first made to contract actively with ACh, and after the maximum isometric tension (Po) is developed, is subject to quick releases to drop the tension to zero at various times after the application and following removal of ACh. Dotted lines indicate time course of tension redevelopment following quick releases. The catch tension is made to relax with 5-HT.

The ABRM is innervated by both cholinergic and serotonergic nerves, releasing acetylcholine (ACh) and 5-hydroxytryptamine (5-HT) from nerve terminals [14,15,16]. The mechanical response of the ABRM fibers is completely different between the active and the catch states. The ABRM fibers can be made to contract actively by cholinergic nerve stimulation or by external application of ACh [17]; when the activated ABRM fibers, producing the maximum isometric tension (Po), are subjected to a sudden decrease in load from Po to P < Po, the fibers shorten actively (Figure 1a). When the fibers are subjected to a quick decrease in fiber length, i.e., a quick release (amplitude, about 5% of its slack length), the tension first falls to zero, and then redevelops actively (Figure 1c). After the cessation of cholinergic nerve stimulation, or after removal of external ACh, the fibers are gradually put into the catch state, during which the isometric tension decays extremely slowly, and the fibers no longer redevelop isometric tension after a quick release (Figure 1b), or no longer shorten actively after the reduction of load from Po to P < Po (Figure 1d) [17]. These features indicate that, during the catch state, the tension is maintained only passively. The transition from the active to the catch state can be observed by recording changes in isometric tension following quick releases, applied at various times after the application and removal of ACh (Figure 2). The extent of tension redevelopment after a quick release decreases with time after removal of ACh, and eventually disappears, indicating the establishment of the catch state. These features can be interpreted as inferring that the development of full active isometric tension (Po) is the prerequisite for the establishment of the catch state. The ABRM fibers in the catch state can be made to relax by serotonergic nerve stimulation or by external application of 5-HT [16,18]. 5-HT produces an increase in the intracellular cyclic AMP (cAMP) concentration [19], which activates cAMP-dependent protein kinase A (PKA) to result in the phosphorylation of a high molecular mass protein, twitchin, terminating the catch state [20].

## 4. Mechanical Response of the ABRM to Quick Increases in Load

**Figure 3:**
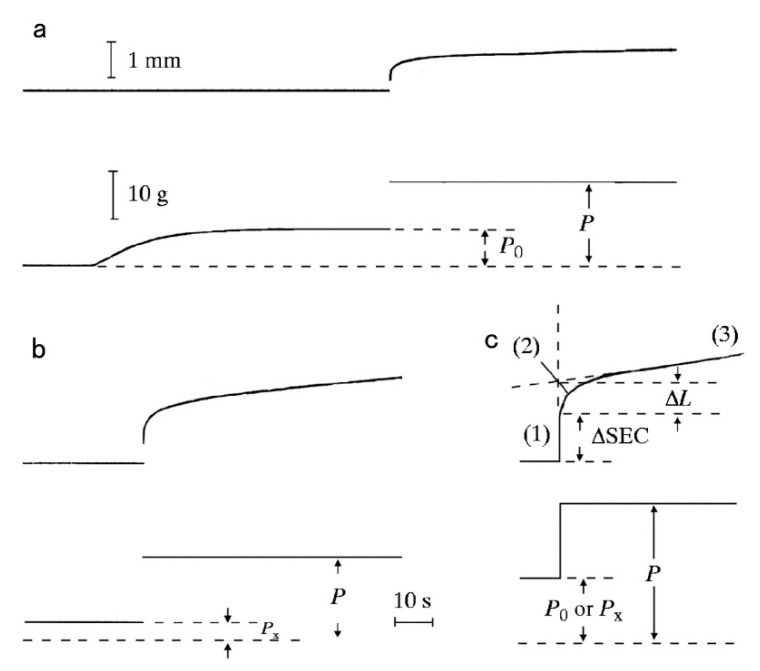
Length (upper traces) and tension (lower traces) changes to quick increases in load in the ABRM fibers producing active tension (Po) (**a**), and during the maintenance of catch tension P_x_ (**b**). The magnitude of quick increase in load was from Po to 2.3 Po in (**a**), and from P_x_ to P = 4.3 P_x_ in (**b**). In (**c**), the early part of the tension change to a quick increase in load, consisting of phases (1) to (3), is illustrated. (Mukou et al. [21]).

**Figure 4:**
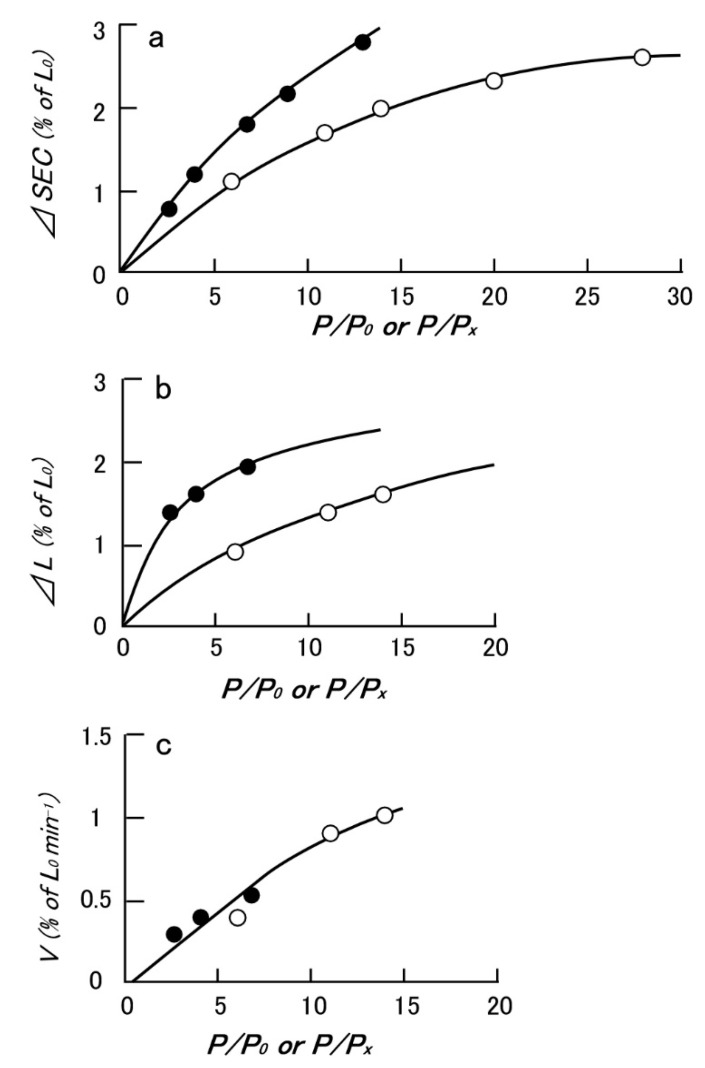
Typical example showing the effect of isotonic load (P/Po or P/P_x_) on the extension of SEC (ΔSEC) (**a**), the distance of early isotonic lengthening (ΔL) (**b**), and the velocity of late isotonic lengthening (**c**). Filled and open circle data points were obtained from the ABRM generating active tension (Po), and maintaining catch tension (P_x_), respectively. (Mukou et al. [21]).

Since the physiological role of the catch state is to resist against external forces (=load) applied to the animal by predators, the mechanical function of the catch state can best be studied by applying step increases in load under various experimental conditions. Mukou et al. [21] compared the time courses of tension responses between the ABRM fibers contracting actively and those in the catch state (Figure 3a,b). The mechanical responses of the ABRM fibers to quick increases in load during the generation of the maximum isometric tension (Po) and during the maintenance of catch tension (P_x_) showed similar time courses, which consisted of three phases (Figure 3c). Namely, (1) the initial quick increase in length coincident with the applied quick increase in load, (2) the early isotonic lengthening, in which the velocity of lengthening decreases rapidly with time, and (3) the subsequent slow isotonic lengthening lasting over many minutes. Phase (1) is regarded as the elastic extension of the series elastic component (SEC), connected in series with the contractile component (CC), and will be called ΔSEC. Meanwhile, Phases (2) and (3) are taken as the early and the late isotonic lengthening of the ABRM, respectively. The distance of the early rapid isotonic lengthening, in which lengthening velocity decreases rapidly with time, will be called ΔL. The values of ΔSEC (expressed as % of the ABRM slack length, Lo), ΔL (expressed as % of Lo), and the velocity of the late isotonic lengthening (V, expressed as % of Lo/min) are dependent on the amount of isotonic load (Figure 4). All data points are obtained from one and the same ABRM preparation during both generations of active tension (Po) (filled circles) and during maintenance of the catch tension (open circles). For a given amount of isotonic load (expressed relative to Po or P_x_), both ΔSEC and ΔL are larger during the active tension generation than during the catch state. On the other hand, the velocity of the late isotonic lengthening (V) was found to fall on the same V as the relative load curve. These features of the mechanical response of the ABRM fibers, especially the quick decrease in lengthening velocity in phase (2), can be taken to indicate that the transition from the active to the catch state is quickened by the sudden application of large loads >Po. Meanwhile, if a load >1.5 Po is applied to the ABRM fibers at the beginning of active tension development, it is lengthened rapidly, like vertebrate skeletal muscle, which is lengthened rapidly under a load >1.8 Po [22].

The results stated above reveal the important function of the catch mechanism in the shell adductor muscle of bivalve molluscan animals. In their normal environment, catch muscle fibers should quickly resist external forces from predators attempting to open the shell. If the establishment of the catch state takes many seconds, as can be observed in the isometric condition (Figure 2), bivalve molluscan animals may not effectively protect themselves against sudden attacks from predators. In this regard, the mechanical response of the ABRM, generating active tension Po (Figure 3a), would be very useful in quickly increasing its resistance against the sudden application of large external forces (=loads) by predators; when the animal becomes aware of attack of predators, it will first fully activate the shell adductor muscle by a brief train of cholinergic nerve impulses, so that they are prepared to rapidly increase their resistance against large external forces, which are suddenly applied even during the generation of active tension (Figure 3a), as well as after the subsequent establishment of the catch state (Figure 3b).

## 5. Translocation of Intracellularly Stored Ca Ions during Transition from the Active to the Catch State

**Figure 5:**
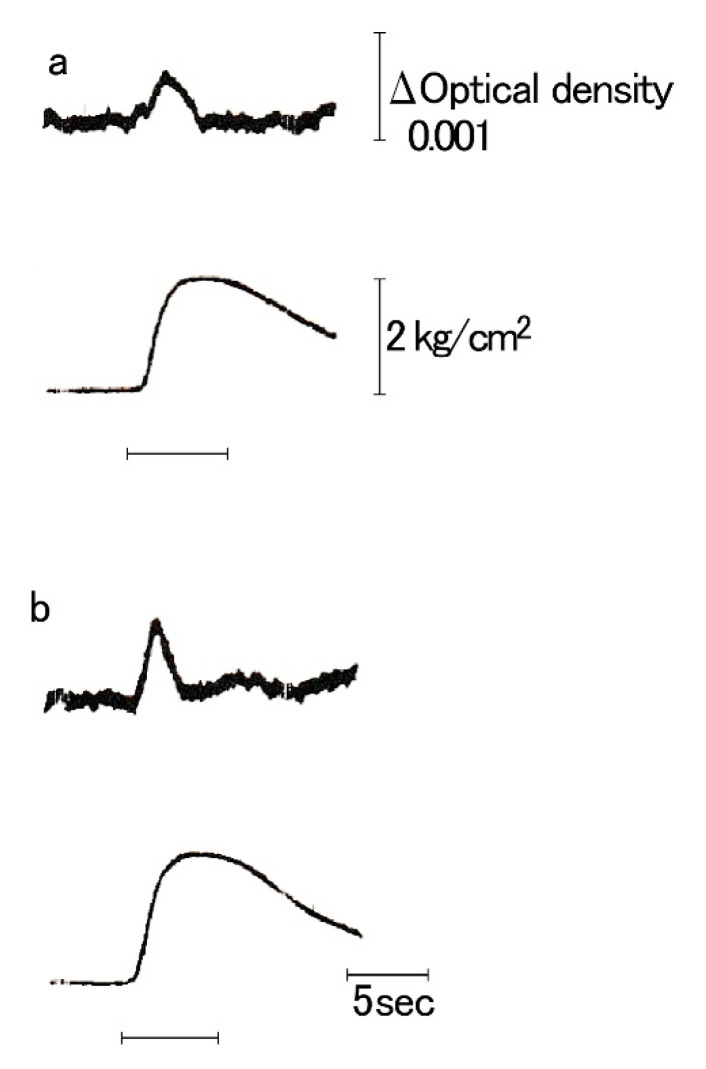
Simultaneous recordings of Ca^2+^ transient (**a**) and isometric tension (**b**) during phasic contraction in the ABRM, elicited by a.c. current (horizontal bar) (Kometani and Sugi, 1978 [21]).

Kometani and Sugi [23] were the first to record intracellular free Ca^2+^ ion concentration [Ca^2+^]i (called the Ca^2+^ transient) in the ABRM using murexide as a Ca^2+^ indicator. During a phasic contraction elicited by the AC current, [Ca^2+^]i increases to a peak during the rising phase of tension, and then starts to decrease to its resting value at the peak of tension (Figure 5). The total area of the Ca^2+^ transient was proportional to the peak tension. These features of the Ca^2*^ transient are qualitatively similar to that of vertebrate striated muscle, indicating that the contractile activity of the ABRM fibers is controlled by [Ca^2+^]i in the same manner as vertebrate skeletal muscle. As the cholinergic nerve releases ACh from its terminals, the increase in [Ca^2+^]i is mediated by ACh. Tameyasu and Sugi [24] showed that ACh did not significantly change Ca^2+^ influx across the cell membrane in the ABRM fibers. Sugi and Yamaguchi [25] also reported that the external Ca^”+^ concentration had almost no effect on the ACh-induced contraction, and that procaine, which is known to prevent Ca^2+^ release from the sarcoplasmic reticulum in vertebrate skeletal muscle, inhibited the ACh-induced contraction in the ABRM fibers. These results are taken to indicate that, in the ABRM fibers, their contractile activity is controlled by the release of intracellularly stored Ca^2+^, but not by Ca^2+^ influx across the cell membrane.

**Figure 6:**
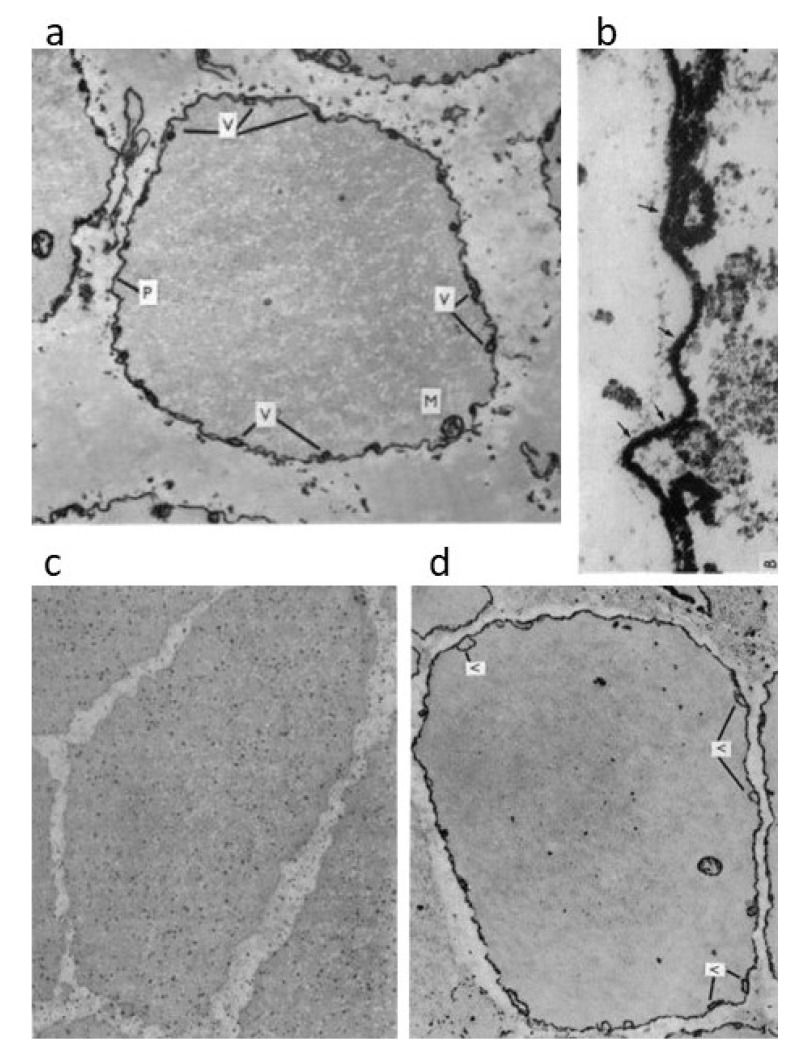
Intracellular localization and translocation of Ca-containing electron-opaque pyroantimonate precipitate in the ABRM fibers. (**a**) Cross-section of resting fibers exhibiting electron-opaque precipitate along the inner surface of the cell membrane (P), at the intracellular vesicles in close apposition to the cell membrane (V), and at the mitochondria (M). (**b**) High magnification view around the cell membrane. Arrows indicate outer surface of the cell membrane. (**c**) Cross-section of actively contracted fibers. Note the diffuse distribution of pyroantimonate precipitates in the cytoplasm in the form of fine particles. (**d**) Cross-sections of fibers in the catch state. Note that pyroantimonate precipitate shows localization similar to that in resting fibers. Calibration, 1 µm for (**a**), (**c**) and (**d**), and 0.1 µm for (**b**). (Atsumi and Sugi [28]).

Using K pyroantimonate, which penetrates intact cell membrane in the presence of Osmium to precipitate Ca^2*^ to produce electron-opaque precipitate with intracellular cations [26,27], Atsumi and Sugi [28] succeeded in visualizing the intracellular translocation of Ca^2+^ in the ABRM fibers electron microscopically. In the resting ABRM fibers, electron opaque pyroantimonate precipitate was observed along the inner surface of the cell membrane, at the vesicles in close apposition to the cell membrane, and at the mitochondria (Figure 6a,b). X-ray microanalysis of the precipitate demonstrated the presence of Ca in the precipitate. In the fibers during active contraction in response to ACh, the Ca-containing precipitate was observed to distribute diffusely in the myoplasm in the form of fine particles (Figure 6c), indicating that Ca^2+^ stored in the intracellular structures is released into the myoplasm to activate the contractile mechanism. In the fibers, put into the catch state after the removal of ACh, Ca-containing precipitate was found to return to the intracellular structures (Figure 6d). These results are consistent with the increase in [Ca^2+^]i in the ABRM fibers upon stimulation (Figure 5), indicating that ACh causes the release of Ca^2+^ from the intracellularly Ca^2+^- accumulating structures, shown in Figure 6a,b, into the myoplasm to induce active tension development. After the removal of ACh, on the other hand, the establishment of the catch state is associated with the return of the released Ca^2+^ from the myoplasm to the intracellular structures. This implies that, in the catch state, [Ca^2+^]i is reduced to a level similar to that in the resting fibers. This idea is supported by Ishii et al. [29], who recorded Ca^2+^ transients in fura-2 loaded ABRM fibers. In accordance with the above electron microscopic findings, Yamanobe and Sugi [30] showed the presence of a high-molecular-mass (450 kDa) Ca^2+^-binding protein in the cell membrane-enriched fraction obtained from the ABRM, and named it MCBP-450. In the lumen of the sarcoplasmic reticulum, on the other hand, a high-molecular-mass Ca^2+^-binding protein, calsequestrin, is present, and is believed to serve as a Ca^2+^ reservoir [31]. Both calsequestrin and MCBP-450 have high Ca^2+^-binding capacity—~38 mol per mol of MCBP-450 and 40–50 mol Ca^2+^ per mol of calsequestrin. It seems therefore possible that, in the ABRM, ACh-induced membrane depolarization decreases the Ca^2+^-binding capacity of MCBP-450 to result in the release of Ca^2+^ from the inner surface of the cell membrane, to activate the contractile mechanism [28].

## 6. Interconnection of the Thick Filaments in the ABRM as a Possible Cause of the Catch State

**Figure 7:**
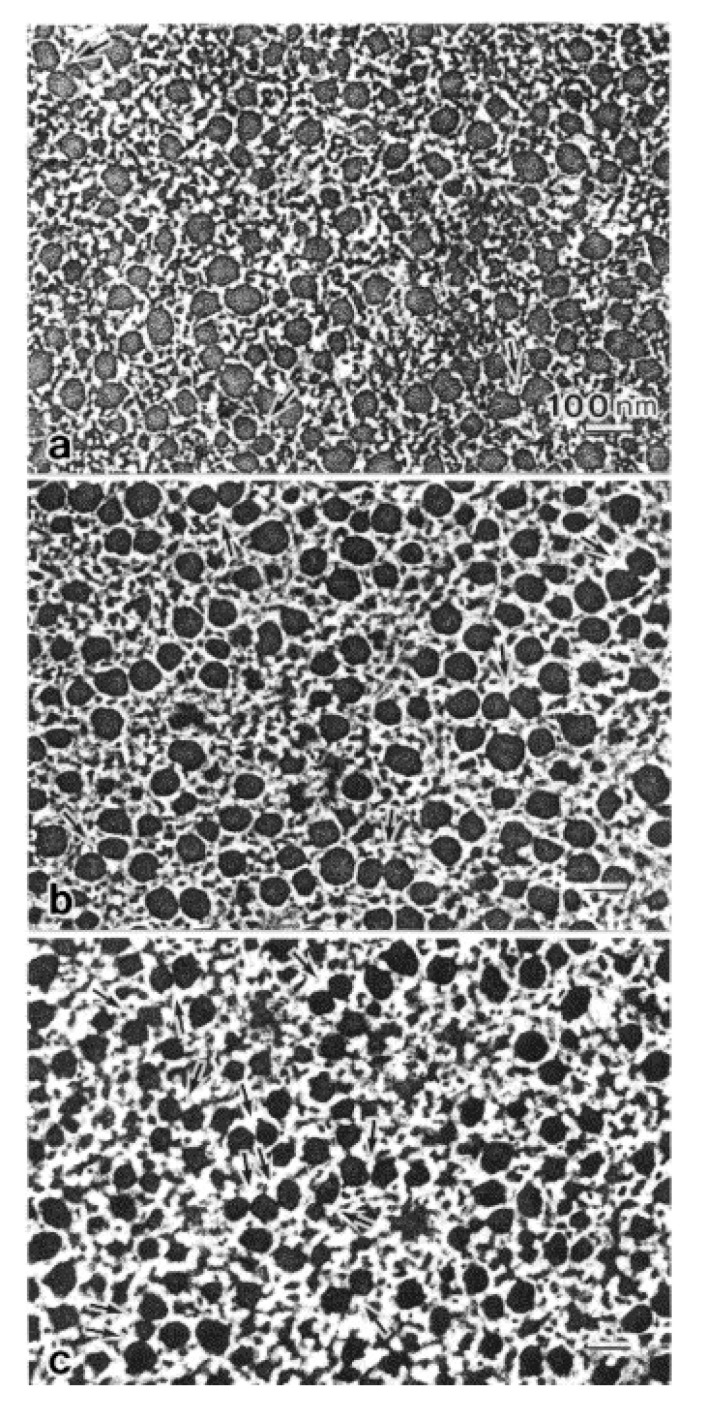
Cross-sections of the ABRM fibers, quickly frozen at rest (**a**), during active tension development (**b**), and during the catch state (**c**). In (**c**), the thick filaments are occasionally interconnected either directly or by projections, as indicated by arrows. Bar, 100 nm. (Takahashi et al. [36]).

**Figure 8:**
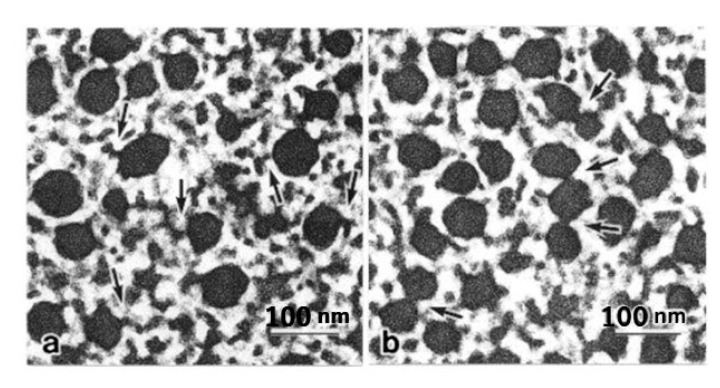
Cross-sections at higher magnification showing linkages between the thick and thin filaments (arrows) in the ABRM frozen during the actively contracting state (**a**), and interconnections between the thick filaments (**b**) in the ABRM frozen during the catch state. (Takahashi et al. [36]).

The marked aggregation of the thick filaments in the ABRM fibers during the catch state has been observed electron microscopically by Heumann and Zebe [32] and Hauk and Achazi [9], suggesting that the catch state is associated with the aggregation of the thick filaments. It has been pointed out, however, that the thick filament aggregation may be an artifact arising from the cross-linking of amino acid residues between adjacent thick filaments, resulting from glutaraldehyde fixation [33,34]. As a matter of fact, Bennett and Elliott [35] examined the ABRM fiber cross-sections after quick freezing and freeze substitution processes, and found no thick filament aggregation. To settle the above conflicting results, Takahashi et al. [36] compared cross-sections of the ABRM fibers, prepared using quick-freezing and freeze substitution techniques (Figure 7). No fusion or aggregation of the thick filaments was observed, not only in the relaxed and the contracting states (Figure 7a,b), but also in the catch state (Figure 7c). This supports the view that the thick filament aggregation is an artifact arising from the cross-linking of amino acid residues in the thick filaments. Meanwhile, in the catch state, the thick filaments were frequently observed to interconnect with each other either directly or by projections (Figure 7c and Figure 8b). It seems possible that the thick filament interconnection is formed by twitchin around the thick filaments [20]. In the actively contracting state, on the other hand, linkages between the thick and thin filaments were frequently observed (Figure 8a), being consistent with the view that active tension development is produced by the cyclic interaction between actin, located in the thin filament, and myosin located in the thick filament.

**Figure 9:**
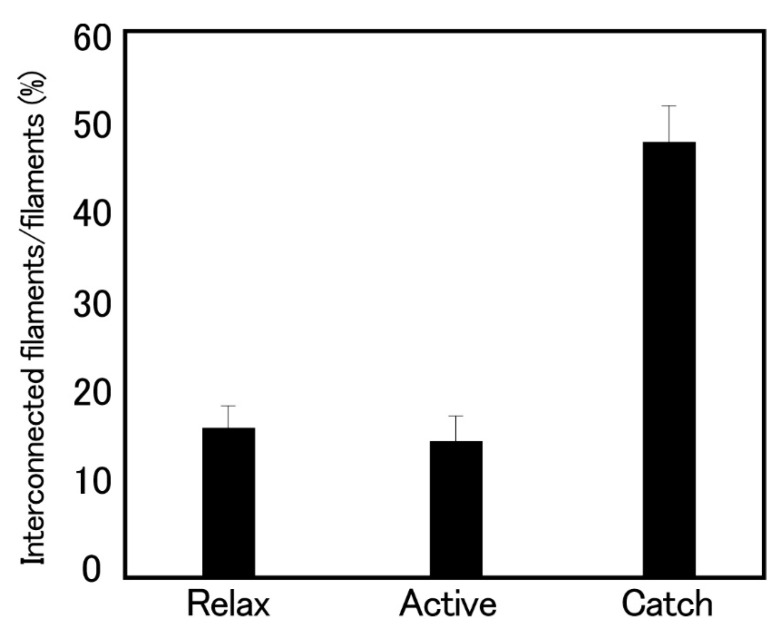
Histograms showing the proportion of the interconnected thick filaments, measured in the cross-sections of the ABRM fibers, quickly frozen in the relaxed, actively contracting and catch states. Bars indicate ±SD (*n* = 8). (Takahashi et al. [36]).

Histograms showing the proportion of the interconnected thick filaments in the ABRM fiber cross-sections prepared at three different states (Figure 9) show the marked increase in the proportion of interconnected thick filaments in the catch state, strongly suggesting that the thick filament interconnection is responsible for the catch state. The reason why Bennett and Elliott failed to observe the thick filament interconnection during the catch state seems to be that they appear to use the whole ABRM instead of small bundles; the resulting incomplete and nonuniform freezing of the ABRM fibers might have led them to reach the wrong conclusion. Close examination of the longitudinal sections of the ABRM fibers in the catch state also indicated both the thick-to-thin and the thick-to-thick interconnections by projections (Figure 9). As already mentioned, the catch state is established only after the removal of ACh, producing the maximum tension (Po) (Figure 2). This indicates that ACh-induced tension development to Po is the necessary prerequisite for the establishment of the catch state. In addition, the mechanical response of the ABRM to quick increases in load (Figure 3 and Figure 4) shows that the catch state establishment is facilitated by applying large external loads, under which the ABRM is lengthened.

## 7. Possible Mechanism of the Catch State Coupled with Intracellular Translocation of Ca^2+^

In the early 1960s, it was generally believed that the catch state is produced by a reduced rate of ADP release from the actin–myosin complex, A-M-ADP-Pi, to result in a reduced rate of tension decay during the catch state [37]. This idea is, however, not supported by the evidence that the catch state is not influenced by inhibitors of actin–myosin interaction [38,39]. In addition, the extremely high resistance against large loads indicates the presence of load-bearing structures other than actin–myosin linkages [21,22]. For the reasons stated above, it may be safe to preclude the possibility that only the actin–myosin linkages are responsible for the catch state. Nevertheless, Yamada et al. [40] have performed in vitro assay experiments, claiming that the catch state results only from actin-–myosin linkages. However, their experimental system consists only of actin and myosin filaments, and differs too much from the ABRM contractile system, in which myosin and twitchin are attached around thick paramyosin filaments. In addition, they completely ignore the possibility that the fusion of thick filaments is involved in the maintenance of catch tension.

It has long been known that the ABRM in the catch state can be relaxed by stimulation of the serotonergic nerve, which releases serotonin (5-hydroxytryptamine, 5-HT) from nerve terminals [19]. It is now clear that the 5-HT-induced relaxation of the catch state is mediated by cyclic AMP(cAMP), which activates protein kinase A to result in the phosphorylation of a high molecular mass (~600 kDa) protein, twitchin [20,41,42]. Butler et al. [43] recently reported that, in skinned ABRM fibers, twitchin phosphorylation increases in stretched fibers, suggesting that twitchin senses tension in the ABRM, to result in an increase in twitchin kinase activation. However, this phenomenon is related to the relaxation of the catch state, but not to the establishment of the catch state.

On the other hand, Funabara et al. [44,45,46] performed extensive biochemical studies of the phosphorylation and dephosphorylation of twitchin, and showed that dephosphorylated twitchin forms a trimeric complex with actin and myosin, indicating that the trimeric complex formation may correspond with the formation of the thick-to-thin filament and the thick-to-thick filament interconnections observed electron microscopically [36]. The fact that the contractile system in the ABRM has both myosin-based and actin-based regulatory mechanisms would certainly be essential for the trimeric complex’s formation, which might well explain the extremely large resistance against large external loads, as shown by Mukou et al. [21].

**Figure 10:**
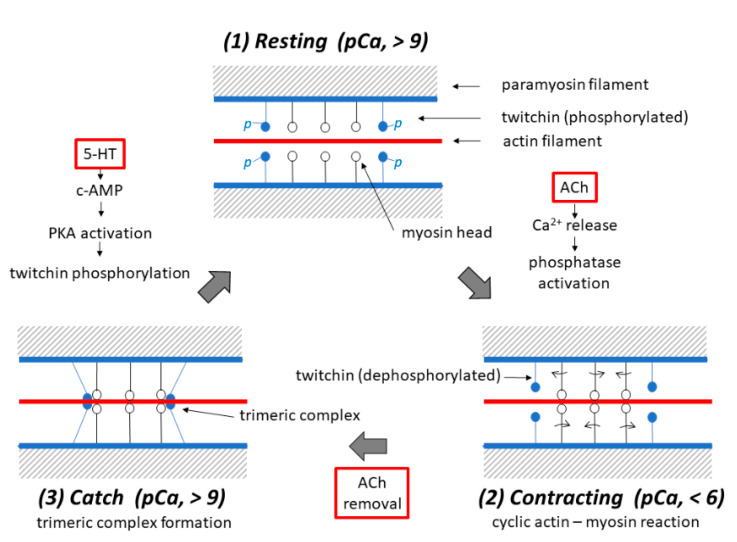
Schematic diagrams illustrating possible mechanisms underlying the three different states, i.e., the (1) resting, (2) contracting and (3) catch states in the ABRM fibers. Each diagram shows a pair of adjacent thick filaments (with a paramyosin core), around which both myosin (with myosin heads extending laterally) and twitchin (with oval-shaped projections colored blue) are bound to face the actin filament (colored red). (1) In the resting ABRM fibers, twitchin is in the phosphorylated state, while t he [Ca^2+^]i in the myoplasm is low (pCa, >9) to inhibit actin–myosin interaction. (2) Upon ACh application, it causes Ca^2+^ release from the intracellular structures, and the resulting increase in [Ca^2+^]i (pCa, <6) produces cyclic actin–myosin interaction to put the ABRM fibers into the contracting state. (3) After ACh removal, [Ca^2+^]i decreases to the resting level due to Ca^2+^ reuptake by the intracellular structures, and dephosphorylated twitchin forms a trimeric complex with actin and myosin to build up the catch state. Application of 5-HT induces c-AMP-dependent protein kinase A (PKA) activation, which in turn causes twitchin phosphorylation to result in the disappearance of the trimeric complex, and the ABRM fibers return to the resting state.

At present, the most plausible explanation for the catch mechanism may be summarized as follows: (1) In the resting ABRM fibers, [Ca^2+^]i is low (pCa, >9), and myosin heads extending from the thick filaments are detached from the actin filaments, while twitchin is in the phosphorylated state, and does not interact with both the actin and myosin filaments. (2) Upon application of ACh, [Ca^2+^]i increases to a high value (pCa, <6) due to the ACh-induced release of Ca^2+^ from the intracellular Ca^2+^-accumulating structures into the myoplasm, to elicit a cyclic interaction between the myosin heads and the actin filaments, i.e., the active contraction of the ABRM fibers, while twitchin is dephosphorylated by Ca^2+^-activated phosphatase. (3) After the ACh, Ca^2+^ in the myoplasm is taken up by the intracellular Ca^2+^-accumulating structures. The resulting reduction in [Ca^2+^]i to the resting level (pCa, >9) makes dephophorylated twitchin form a trimeric complex with actin and myosin, to build up the catch state, during which the ABRM fibers exhibit extremely large resistance against external loads. (4) Upon application of 5-HT, which causes the c-AMP-dependent activation of protein kinase A to phosphorylate twitchin, the trimeric complex disappears, to result in the relaxation of the catch state. Figure 10 shows diagrams illustrating the sequence of events, producing the three different states in the ABRM. Of course, these diagrams are at present tentative and incomplete. Much more experimental work is necessary to reach a full understanding of the catch mechanism. One possible approach may be, for example, to observe the time course of filament interconnections and fusions electron microscopically, using the technique of the quick-freezing of ABRM fibers, and corelating the results with the development of the catch tension. As already mentioned, progress in the study of the catch state would contribute to an understanding of the latch state in the vascular smooth muscle.
